# Anti-MRSA Activities of Enterocins DD28 and DD93 and Evidences on Their Role in the Inhibition of Biofilm Formation

**DOI:** 10.3389/fmicb.2016.00817

**Published:** 2016-05-31

**Authors:** Ahmed K. Al Atya, Yanath Belguesmia, Gabrielle Chataigne, Rozenn Ravallec, Anne Vachée, Sabine Szunerits, Rabah Boukherroub, Djamel Drider

**Affiliations:** ^1^Université de Lille 1 Sciences et Technologies – Institut Charles Viollette Lille, France; ^2^Hôpital Victor Provo de Roubaix Roubaix, France; ^3^Institut d’Electronique, de Microélectronique et de Nanotechnologie, UMR CNRS 8520, Université Lille 1 Lille, France

**Keywords:** enterocins, antibiotics, MRSA, synergism, bacteriocins

## Abstract

Methicillin-resistant *Staphylococcus aureus* (MRSA) has become a worrisome superbug. This work aimed at studying the effects of two class IIb bacteriocins, enterocins DD28 and DD93 as anti-MRSA agents. Thus, these bacteriocins were purified, from the cultures supernatants of *Enterococcus faecalis* 28 and 93, using a simplified purification procedure consisting in a cation exchange chromatography and a reversed-phase high-performance liquid chromatography. The anti-Staphylococcal activity was shown *in vitro* by the assessment of the minimal inhibitory concentration (MIC), followed by a checkerboard and time-kill kinetics experiments. The data unveiled a clear synergistic effect of enterocins DD28 and DD93 in combination with erythromycin or kanamycin against the clinical MRSA-S1 strain. Besides, these combinations impeded as well the MRSA-S1 clinical strain to setup biofilms on stainless steel and glace devices.

## Introduction

*Staphylococcus aureus* is among the five top pathogens found as normal resident of the skin and nasal flora in at least 25–30% of healthy humans, and it is associated with hospital acquired (HA-MRSA) and community acquired (CA-MRSA) infections ranging from superficial wound infections to life-threatening deep infections such as septicemia, endocarditis, and toxic shock syndrome (David and Daum, 2010). Antibiotic resistance and biofilm-forming capabilities contribute to the success of *S. aureus* as a harsh human pathogen in the healthcare as well as in the community settings. The last decade has seen a welcome increase in the number of agents available for the treatment of MRSA including antibiotics such as fluoroquinolones, linezolid, rifampin, and antimicrobial peptides (AMPs) such as daptomycin, tigecycline, and mainly vancomycin. Resistance to methicillin was observed in 1961, 1 year after the commercial availability of this antibiotic. Susceptibility to vancomycin was first reported in 1996 in Japan, leading to emergence of heterogeneous resistance to vancomycin phenotype (Spagnolo et al., 2014). MRSA with reduced susceptibility to vancomycin was reported in ocular infections, and there was a rise in *S. aureus* resistance to new and old generation fluoroquinolones that were commonly used for prophylaxis after intravitreal injections and intraocular surgeries (Sadaka et al., 2015). Daptomycin which is considered drug of last resort after vancomycin breakdown for the treatment of MRSA infections (Claeys et al., 2015) has shown non-inferiority to vancomycin in the treatment of MRSA bacteremia (Holmes et al., 2015) was threatened because of the emergence of daptomycin resistance, especially in the deep-seated infections (Claeys et al., 2015). MRSA are responsible of diverse infections, especially in the healthcare structures. The increasing resistance of Gram-positive bacteria to the broad-spectrum antibiotics and the lack of new molecules expected to become available in the near future, advocates the need of novel anti-MRSA agents and therapeutic options (Tängdén, 2014).

Antimicrobial peptides were largely admitted as potential alternatives to traditional antibiotics in order to combat the scaring and increasing bacterial infections. AMPs are produced by all the living cells but also gathered by chemical synthesis and controlled enzymatic digestion of proteins. Bacteria are known as great sources of AMPs such as lipopeptides and bacteriocins. Conversely to lipopeptides, the bacteriocins are AMPs of proteinaceous nature, ribosomally synthesized mainly by lactic acid bacteria (LAB) (Drider and Rebuffat, 2011). LAB of *Enterococcus* genus produce a great number of bacteriocins designed as enterocins. Enterocins-producing strains were isolated from a wide range of sources, including fermented food, environmental, and clinical (Ishibashi et al., 2012). Enterocins resulted to be mainly produced by *Enterococcus faecalis* and *E. faecium* species (Goto and Yan, 2011). Enterocins produced by *E. muntii*, *E. avium, E. durans*, and *E. hirae* strains were also reported in the literature (Saavedra et al., 2004; Batdorj et al., 2006; Sánchez et al., 2007; Birri et al., 2010). Multiple enterocins-producing strains were characterized for their large range of activities, inhibiting the growth of many undesirable bacteria (Ishibashi et al., 2012). Cintas et al. (2000) underlined the potential of *E. faecium* L50 to produce three different enterocins named enterocins L50A and L50B, enterocin P, and enterocin Q which act synergistically and inhibit the growth of many Gram-positive bacteria. Remarkably, enterocins were also produced by enterococci from the gastrointestinal tract origins of humans, animals, human infection sites and healthy babies feces (Tomita et al., 1996; Kang and Lee, 2005; Sawa et al., 2012) A compilation of studies underpinning the inhibitory activities of enterocins, pointed out the capabilities of enterocin E-760 to inhibit the growth of *Salmonella enterica*, *Escherichia coli*, *Pseudomonas aeruginosa*, *Campylobacter jejuni*, and *Staphylococcus aureus* (Line et al., 2008). These data show that bacteriocins are sustainable antimicrobials that could be used alone or in combination with antibiotics.

Related to that, the combinations of antibiotics were presented as a promising option in the management of antibiotics use and treatment of life-threatening infections (Bassetti and Righi, 2015; Leone et al., 2015). The antibiotics were successfully combined to chemical or physical agents (Sawa et al., 2012; Kullar et al., 2015). In direct line, the combinations of antibiotics and bacteriocins could offer novel therapeutic options as supported by different and successful *in vitro* studies (Naghmouchi et al., 2010, 2011, 2012, 2013; Hassan et al., 2012; Mataraci and Dosler, 2012; Wolska et al., 2012; Singh et al., 2014).

In this study, enterocins DD28 and DD93 produced by *E. faecalis* 28 and *E. faecalis* 93 were purified and their DNA sequences were determined. The anti-MRSA activity was determined against the MRSA-S1 clinical strain. In spite of their relatively high MICs values against the aforementioned target, enterocins DD28 and DD93 were able to synergize with kanamycin or erythromycin and switch from resistance to susceptibility the phenotype of MRSA-S1 strain. Furthermore, these combinations hampered biofilm formation of the MRSA-S1 strain on AISI 304L stainless steel and glass devices. Overall, the combinations of antibiotics and bacteriocins offer a novel strategy to fight against pathogenic bacteria.

## Materials and Methods

### Strains, Cultivation, and Antibacterial Susceptibility

*Enterococcus faecalis* DD28 and *E. faecalis* DD93 recently isolated from meconium (Al-Atya et al., 2015) were grown in MRS (de Man, Rogosa, and Sharpe) medium (De Man et al., 1960) at 37°C for 16 to 18 h. *S. epidermidis* (Kindly provided by Dr. Anne Vachée, Roubaix hospital, France), *S. aureus* ATCC 33862, and Methicillin Resistant *S. aureus* (MRSA) strains including MRSA ATCC 43300, MRSA-S1, and MRSA-2 strains (kindly provided by Dr. Gilles Prévost, Strasbourg University, France) were grown in Brain Heart Infusion (BHI) broth at 37°C for appropriate experimental time. *Listeria innocua* CIP80.11 was grown at 37°C for overnight in BHI or Muller Hinton media according to the experiment purposes.

Antibiotic susceptibility was performed by VITEK 2 system (Bio-Mérieux, France), encompassing nearly all important antibiotics. Antibiotic susceptibility and MICs were determined and analyzed according to the French Committee on Antimicrobial Susceptibility Testing (French Committee on Antimicrobial Susceptibility Testing [FCAST], 2015).

### Purification of Bacteriocins and Determination of Their Masses

Purification of enterocins DD28 and DD93 was performed at room temperature using two-step methods adapted from Abriouel et al. (2003). Briefly 1,000 ml of the cell-free culture supernatant of *E. faecalis* 28 and *E. faecalis* 93 were adjusted to pH 6.3 using 5 M NaOH. Then Carboxymethyl Sephadex CM-25 (GE Healthcare, Sweden) gel slurry equilibrated in distilled water was added to culture supernatants (1:40, v/v). The obtained mixture was held under stirring for 30 min and decanted for additional 30 min. The supernatants were removed, and the sediment gel slurry containing the bacteriocin activity, captured by cation interactions with the matrix was loaded onto a 10 cm × 50 cm glass column. The gels was washed with one volume (1V) of distilled water and 2V of 0.5 M NaCl, followed by 2V of 1.5 M NaCl to elute the adsorbed bacteriocin. The obtained fractions were filtrated through 0.22 μm pore size low protein binding filters (Millex GV; Millipore Corp., Belford, MA, USA) and tested for bacteriocin activity.

The active fractions from the cation exchange chromato-graphy were applied onto a reversed-phase high-performance liquid chromatography (RP-HPLC) by using a column C-18 (5 μm, 250 mm × 3 mm, VYDAC 218 TP53; Grace, Deerfield, IL, USA) previously equilibrated in solvent A (10 mM trifluoroacetic acid, TFA; Fluka), at a flow rate of 5 ml/min. Non-adsorbed material was eliminated by washing the column with solvent A until the UV absorbance of the effluent at 210 nm reached baseline. The material retained in the column was eluted with a gradient ranging from 0 to 40% of solvent B [isopropyl alcohol/acetonitrile (2:1, v/v) in 40 mM TFA] in over 5 min, then followed by increasing of solvent B gradient from 40 to 100% for over 25 min at a flow rate of 2.5 ml/min. Fractions of the column effluent were collected according to their UV light absorbance, lyophilized and dissolved again in distilled water before being tested for bacteriocin activity.

Antimicrobial activity was tested along the purification procedure on the cell-free supernatant (CFS), partly purified peptide (upon cation exchange step) and purified peptide (upon Reversed Phase-HPLC step). Arbitrary units (AU) were calculated based on the spot method (Naghmouchi et al., 2011). Thus, the resulting sample was serially diluted twofold with filter-sterilized phosphate buffer. Ten micro liter of each diluted sample was spotted onto the plate of BHI medium containing *L. innocua* CIP80.11 as indictor strain. The plates were then incubated at 37^o^C overnight, and the titer was defined as the reciprocal of the highest dilution (2*^n^*) that resulted in inhibition of the indicator lawn. The AU of antibacterial activity per milliliter was defined as 2*^n^* × 1,000 μl/10μl. The protein concentration was determined by BCA assay using Sigma–Aldrich BCA kit (USA).

Purified enterocins were analyzed with an Ultraflex MALDI-ToF/ToF mass spectrometer (Bruker, Bremen, Germany) equipped with a smart beam laser. Samples were analyzed using an accelerating voltage of 25 kV and matrix suppression in deflexion mode at m/z 1000. The laser power was set to just above the threshold of ionization (around 60%). Spectra were acquired in reflector positive mode in the range of 3 000 at 10 000 Da. Each spectrum was the result of 1 000 laser shots per m/z segment per sample delivered in 10 sets of 50 shots distributed in random locations on the surface of the matrix spot. The instrument was externally calibrated in positive reflector mode using Bradykinin (1–7) [M+H]^+^ 757.3991, Angiotensin II [M+H]^+^ 1 046.5418, Angiotensin I [M+H]^+^ 1 296.6848, Substance P [M+H]+ 1347.7354, Bombesin [M+H]^+^ 1619.8223, ACTH (1–17) [M+H]^+^ 2 093,0862. For analysis, a mixture of 1 μl of purified enterocin and 1 μl of α-cyano-4-hydroxycinnamic acid (10 mg/ml 70:30 water/acetonitrile with 0.1% TFA) was spotted onto a MALDI-TOF MTP 384 target plate (Bruker Daltonik GmbH, Leipzig, Germany) according to the procedure of the dried-droplet preparation.

### Minimal Inhibitory Concentrations (MICs) and Checkerboard Experiments

A pure colony of each *Staphylococcus* strains used in this study was grown overnight in BHI medium at 37°C. Afterward 10 μl of each overnight culture were added to the wells of bioassay microplates of 96 well cell culture plate (Cellstar) containing different concentrations of enterocins DD28 and DD93, ranging from 50 to 800 mg/l of each bacteriocin. The minimal inhibitory concentration (MIC) is defined as the lowest concentration of an antibiotic that will inhibit the visible growth of a microorganism after overnight incubation.

Antimicrobial agent interactions were determined using checkerboard assay. The concentrations used for enterocins DD28 and DD93 were comprised between 25 and 400 mg/l, while those used for erythromycin and kanamycin were ranging from 0.25 to 64 mg/l. Microplates were inoculated with MRSA-S1 strain at about 10^6^ CFU/ml, in a final volume of 200 μl per well, and incubated overnight at 37°C. The fractional inhibitory concentration index (FICI) was calculated for each combination using the following formula: FICA + FICB = FICI, where FICA = MIC of drug A in combination/MIC of drug A alone, and FICB = MIC of drug B in combination/MIC of drug B alone. The FICI was interpreted as follow: synergism = FICI ≤ 0.5; indifference = 0.5 < FICI ≤ 4; antagonism = FICI > 4 (Petersen et al., 2006).

### Killing Curves Experiment

This experiment was realized on the MRSA strain. Tubes containing BHI supplemented with enterocin DD28, enterocin DD93, erythromycin, kanamycin or combination of bacteriocin and antibiotic, at previously defined concentrations during checkboard assay, were inoculated with MRSA-S1 strain to a density of about 5 × 10^5^ CFU/ml in a final volume of 5 ml and incubated at 37°C for 24 h. The killing kinetics of the enterocin DD28 alone, enterocin DD93 alone, erythromycin alone, kanamycin alone and enterocin DD28 or enterocin DD93 in combination with erythromycin or kanamycin was assessed against MRSA-S1 strain using standard time-killing experiments and viable bacterial counts on agar plates. The final concentration of enterocin DD28 and enterocin DD93 was 50 mg/l, erythromycin 1 mg/l, kanamycin 4 mg/l and for combination of bacteriocins with antibiotics the concentrations used were 50 mg/l for enterocin DD28 and DD93 and 1 mg/l or 4 mg/l, respectively, for erythromycin and kanamycin.

Aliquots were removed at different times 0, 3, 6, 9, and 24 h of incubation, and then serially diluted in saline solution for determination of viable counts. Diluted samples (100 μl) were plated on Tryptone Soya Agar (TSA) plates and colonies were counted after overnight incubation at 37°C. Bactericidal activity was determined as 3 log_10_ CFU/ml reduction in the colony count relative to the initial inoculums (Pankuch et al., 1994).

### DNA Extraction and PCR Amplification of Enterocins Codifying DNA

Total DNA from *E*. *faecalis* 28 and *E. faecalis* 93 were extracted following the same procedure recently described by Al-Atya et al. (2015). The following forward: ATGGGAGCAATCGCAAAATTAGTAG and reverse: TTAATGTCTTTTTAGCCATTTTTCAATTTG primers (Liu et al., 2011) were used to amplify the genes encoding enterocin L50A and L50B. The PCR conditions consisted in: initial denaturing step of 5 min at 95°C, followed by 30 cycles of 1 min denaturing at 95°C, 1 min annealing at a temperature specific for the primers for each of the known enterocin gene, and 10 min extension at 72°C. Polymerase chain reaction (PCR) was done using the PCR Master Mix (2X) (Thermo Scientific Fermentas, Villebon sur Yvette, France) as a mixture of Taq DNA polymerase. DNA extraction was performed using the Wizard^®^ Genomic DNA Purification Kit (Promega Corp., Madison, WI, USA). Ligation of PCR products was done into pGEM-T Easy vector (Promega Corp., Madison, WI, USA). Plasmid extraction was carried out using GeneJET Plasmid DNA Purification Kit (Thermo Scientific Fermentas). Restriction endonucleases were supplied by Thermo Scientific Fermentas. Ligation of inserts to different vectors was effected using the DNA Ligation Kit < Mighty Mix > from Takara (Ozyme, Saint Quentin en Yvelines, France). Recovery of DNA from agarose gels was performed with GeneJET Gel Extraction kit (Thermo Scientific Fermentas).

In all cases, the instructions of the suppliers were followed. All the construction sequences were checked by DNA sequencing performed at Eurofins MWG operon (Ebersberg, Germany).

### Biofunctionalization of AISI 304L Stainless Steel and Glass Slides with Antimicrobial Compounds

To study the effect of treatment of stainless steel slide, reproducing abiotic surfaces used in healthcare units and industries environment, we used the protocol adapted from Ait Ouali et al. (2014). Briefly 2 ml of antimicrobials at their MIC values: enterocin DD28 (200 mg/l), enterocin DD93 (200 mg/l), erythromycin (8 mg/l), ofloxacin (0.5 mg/l), vancomycin (1 mg/l), rifampin (0.03 mg/l), and combination of enterocin DD28 with erythromycin (50/1 mg/l) were added on the surface of each AISI 304L slide placed in sterile Petri plates, and incubated for 2 h at 37°C. During this step a conditioning film on the AISI surface may be formed. Sterile Tryptone Soy Broth – Yeast Extract (TSB-YE) was added in an additional AISI304L slide as a control for this conditioning step. After this step the antimicrobial compounds and the TSB-YE were removed and replaced by 2 ml of MRSA-S1 strain suspension at 10^7^ CFU/ml. After 1 h of incubation time, the supernatant containing non-adherent MRSA-S1 strain cells was removed and replaced by 2 ml of sterile TSB-YE medium on the surface of each slide, and the incubation was conducted for 0, 3, 6, and 24 h to survey the installation of MRSA-S1 strain biofilm. After each time of incubation, the slides were washed twice with 30 ml of Phosphate Buffered Saline (PBS) (pH 7.0). Finally, the AISI 304L stainless steel slides were immersed individually in 30 ml phosphate buffer and sonicated. The detached MRSA-S1 cells were enumerated by plating the bacteria on TSA after growth at 37 °C for 24 h. Additional slides were prepared and served for epifluorescence observation after their staining with live/dead components as explained below.

For scanning electron microscope (SEM) analysis, sterilized glass slides of 1 cm^2^ were deposited in wells of sterile 24-well tissue culture plates (BD Falcon, USA). Then, as previously, the glass slides were treated with 2 ml of these following antimicrobial compounds : enterocin DD28 (200 mg/l), vancomycin (1 mg/l) and combination of enterocin DD28 with erythromycin (50/1 mg/l), or TSB-YE medium as control, during 2 h at 37°C. After this conditioning step of the glass slides, the antimicrobial compounds solutions were removed and replaced by 2 ml of MRSA suspension at 10^7^ CFU/ml. The following steps are exactly the same applied on the AISI304L stainless steel slides; however, incubation was conducted for 5 consecutive days, changing the TSB-YE medium each 24 h. Once the incubation ended, the glass slides were prepared for SEM analysis.

### Epifluorescence and SEM Microscopy Analyses

Biofilm cells were stained with a BacLight LIVE/DEAD bacterial viability staining kit according to the manufacturer’s instructions (Molecular Probes, Invitrogen, France). After dilution of 1.5 μl of each reagent with 1 ml of physiological water (0.85% m/v NaCl), the obtained mixture was gently deposited on the upper face of the slide where the biofilm development may occurred. Following the incubation (15 min in the dark) of the slides, the staining solution was aspirated and biofilms were observed using an epifluorescence microscope (Nikon Optiphot-2 EFD3, Japan).

## Results

### Assessment of Enterocins DD28 and DD93 Amounts upon Purification Process

**FIGURE 1:**
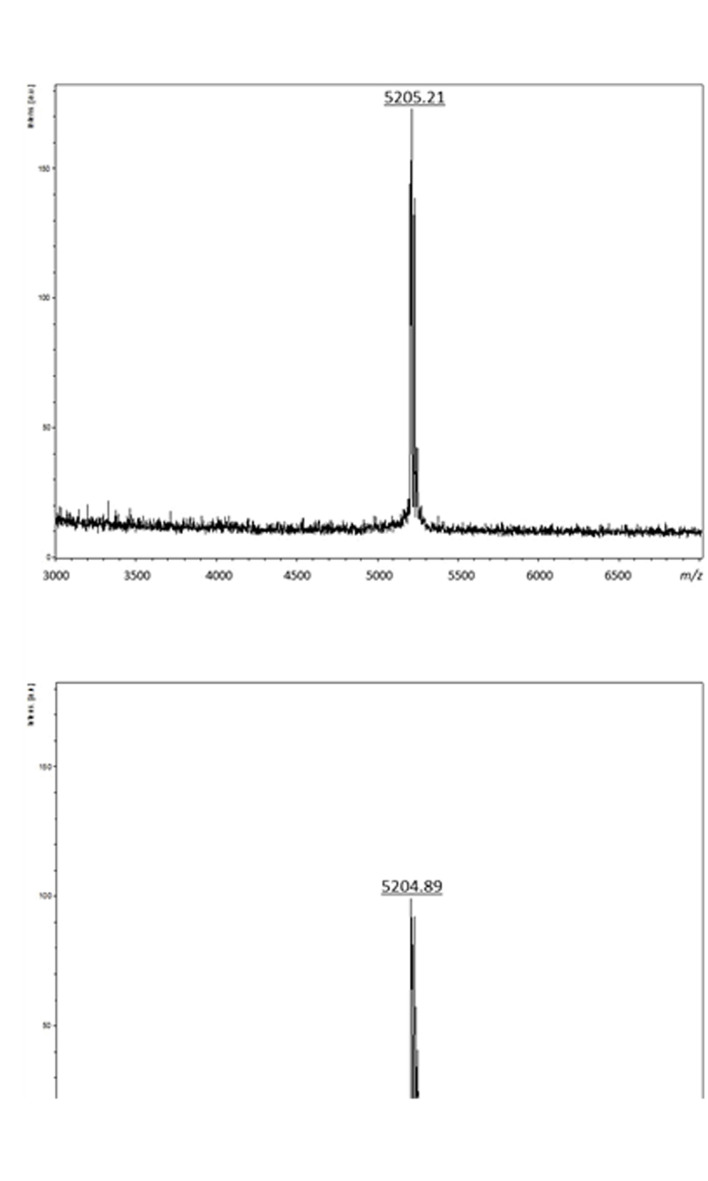
**Mass spectrometry analyses of purified enterocin DD28 **(A)** and enterocin DD93 **(B)****.

The first step of the purification procedure permitted to enhance the specific activity recovered from the CFS, from 86.20 to 3,902.44 AU/mg (enterocin DD28) and 96.15 to 2,318.84 AU/mg (enterocin DD93) (**Table 1**). The second purification step involving a separation on RP-HPLC column permitted to purify these enterocins to homogeneity and evaluate their runtime to 42.5 min. Importantly, the specific activity has increased to 67,368.42 AU/mg for enterocin DD28 and 77,108.43 AU/mg for enterocin DD93 (**Table 1**). The purified enterocins DD28 and DD93 were analyzed by mass spectrometry and appeared to have very close molecular masses of, respectively, 5,205.21 Da and 5,204.89 Da (**Figure 1**).

**Table 1:** Purification of Enterocins DD 28 and DD93.

Strains	Purification stages	Supernatant volume (ml)	Activity AU/ml	Protein (mg/ml)	Total protein (mg)	Total activity	Specific activity (AU/mg)	Purification factor
*Enterococcus faecalis* 28	Supernatant	1 000	800	9.28	9 280	800 000	86.20	1
	Sephadex CM 25	50	3 200	0.82	41	160 000	3 902.44	45.27
	C18 RP-HPLC	1	6 400	0.095	0.095	6 400	67 368.42	781.53
*E. faecalis* 93	Supernatant	1 000	800	8.32	8 320	800 000	96.15	1
	Sephadex CM 25	50	1 600	0.69	34.5	80 000	2 318.84	24.11
	C18 RP-HPLC	1	6 400	0.083	0.083	6 400	77 108.43	801.96


*Protein concentration was determined by BiCinchoninic acid Assay (BCA) kit and the manufacturer recommendations (Sigma–Aldrich, USA). Antibacterial activity was measured against *L. innocua* CIP80.11 as indictor strain.*

### Enterocins DD28 and DD93 were Active against *Staphylococci*

Different *S. aureus* strains were used to demonstrate the enterocins antibacterial activity. Thus strains *S. epidermidis*, *S. aureus* ATCC 33862, MRSA ATCC 43300, MRSA-S1, and MRSA-S2 were used as target strains. The MICs values were similar for *S*. *epidermidis* and *S. aureus* ATCC 33862, but they were twice higher for MRSA ATCC 43300, MRSA-S1 and MRSA-S2 strains. For this study, we have then proceeded with MRSA-S1 strain that was previously isolated from the blood of an 83 years old patient and studied for its susceptibility to chromagranin-derived peptides (Aslam et al., 2013).

### Antibiotics Resistance of MRSA-S1 Clinical Strain

The susceptibility of MRSA-S1 strain as determined by VITEK2 method showed resistance to erythromycin and kanamycin with MICs values of ≥8 mg/l and 32 mg/l, respectively. In turns, this strain exhibited sensibility to ofloxacin (0.5 mg/l), rifampin (0.03 mg/l), and vancomycin (1 mg/l) (**Table 2**). Notably, both enterocins DD28 and DD93 showed inhibition against MRSA-S1 strain and *S. aureus* ATCC 33862 used as controls (**Table 3**). The MICs values were 100 mg/l for *S. aureus* ATCC 33862, and 200 μg/ml for MRSA-S1 strain (**Table 3**).

**Table 2:** Antibiotic susceptibility of MRSA-S1 strain.

Antibiotics	MIC (mg/l)	Antibiotics	MIC (mg/l)
Benzylpenicillin	R 0.25	Linezolid	S 2
Oxacillin	R 1	Teicoplanin	S ≤ 0.5
Gentamicin	S ≤ 0.5	Vancomycin	S 1
Kanamycin	R 32	Tetracyclin	S ≤ 1
Tobramycin	R ≥ 16	Fosfomycin	S ≤ 8
Ofloxacin	S ≤ 0.5	Nitrofurantoin	S ≤ 16
Erythromycin	R ≥ 8	Fusidic acid	S ≤ 0.5
Lincomycin	R ≥ 16	Rifampin	S ≤ 0.03
Pristinamycin	S 1	Trimethoprim-sulfamethoxazole	S ≤ 10


*R, Resistant; S, Sensitive; MIC, Minimal inhibitory concentration determined by Vitek 2 system.*

**Table 3:** Determination of minimum inhibitory concentration (MIC).

Strains	MIC (mg/l) of Enterocin DD28	MIC (mg/l) of Enterocin DD93
*Staphylococcus aureus* ATCC33862	100	100
*Staphylococcus epidermidis*	100	100
MRSAATCC 43300	200	200
MRSA-S1 strain	200	200
MRSA-S2 strain	200	200


### Checkerboard Assays Revealed a Synergistic Effect of Enterocins DD28 and DD93 with Antibiotics

When erythromycin and kanamycin were used in combination with enterocin DD28 or enterocin DD93, the MICs values were lower than those obtained when these molecules were tested individually. These combinations permitted a synergetic effect regarding the FIC values of 0.31 registered for both enterocins DD28 and DD93, in combination with erythromycin and kanamycin on MRSA (**Table 4**). As evidenced from this assay, the MICs values of erythromycin and kanamycin have dropped under their breakpoint points (EUCAST, 2016) when they were associated to enterocins DD28 and DD93.

**Table 4:** Effects of antimicrobials combinations against MRSA-S1 strain.

Strain	DD28 (mg/l)	Kanamycin (mg/l)	Erythromycin (mg/l)	DD28- Kanamycin (mg/l)	FIC	DD28- Erythromycin (mg/l)	FIC
MRSA-S1	200	32	16	50/4	0.375	50/1	0.31

	**DD93 (mg/l)**	**Kanamycin (mg/l)**	**Erythromycin (mg/l)**	**DD93- Kanamycin (mg/l)**	**FIC**	**DD93- Erythromycin (mg/l)**	**FIC**

MRSA-S1	200	32	16	50/4	0.375	50/1	0.31


*Fractional Inhibitory Concentration (FIC) index. The data (±SD) are the average of at least three independent experiments. (MIC are given in mg/l). Kanamycin sulfate (K4000-5G) and Erythromycin (E 5389-5G) were obtained from Sigma–Aldrich (USA).*

### Killing Curves Kinetics Confirmed the Synergistic Effects

**FIGURE 2:**
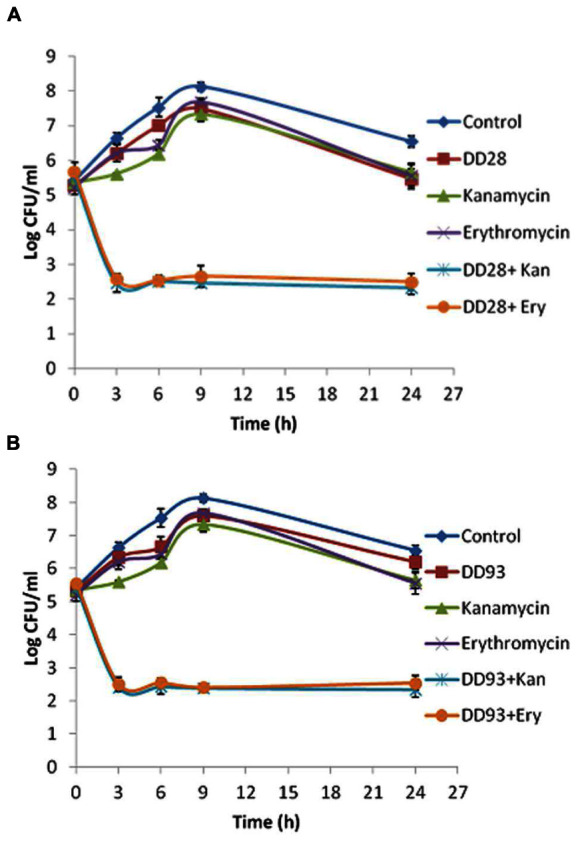
**Time-killing kinetics on planktonic cultures of MRSA-S1 strain at 0, 3, 6, 9, and 24 h in the presence of enterocins DD28 and DD93 (50 mg/l) alone or combined with kanamycin (4 mg/l) and erythromycin (1 mg/l) against SARM-S1.** **(A)** Shows the effect of enterocin DD28+Kanamycin at 50 mg/l-4 mg/l and enterocin DD28+erythromycin at 50-1 mg/l against MRSA-S1. **(B)** Shows the effect of enterocin DD93+kanamycin (50-4 mg/l) and enterocin DD93+erythromycin (50-1 mg/l) against MRSA-S1. The data (±SD) are the average of at least three independent experiments. In each experiment, three measures were performed.

The killing curves experiments realized on MRSA-S1 strain treated with erythromycin and kanamycin and their combinations with enterocins DD28 or DD93 have confirmed the synergistic effect anticipated by the FIC values. As expected, the aforementioned antibiotics are devoid of any inhibitory activity against the planktonic MRSA-S1 culture. The population number as means of CFU/ml remained identical in the untreated samples, as well as in those treated with erythromycin and kanamycin alone portraying this lack of activity. Nevertheless, the combination of enterocins DD28 and DD93 with erythromycin and kanamycin reduced the CFU/ml counts of MRSA-S1 by at least 2–3 logs during 3–24 h of incubation, leading to a killing of 99–99.9% of the CFU/ml of the initial bacterial populations (**Figure 2**; **Table 5**).

**Table 5:** Effect of DD28-Kanamycin, DD28-Erythromycin, DD93-Kanamycin, and DD93-Kanamycin combination against MRSA-S1.

Combination	0 h	3 h	6 h	9 h	24 h
Control	5.41 ± 0.08	6.64 ± 0.15	7.52 ± 0.26	8.12 ± 0.12	6.54 ± 0.16
DD28-Kanamycin (50-4 mg/l)	5.52 ± 0.14	2.45 ± 0.26	2.52 ± 0.15	2.47 ± 0.12	2.33 ± 0.20
DD28-Erythromycin (50-1 mg/l)	5.6 ± 0.26	2.58 ± 0.05	2.53 ± 0.07	2.65 ± 0.32	2.49 ± 0.24
DD93-Kanamycin (50-4 mg/l)	5.42 ± 0.03	2.41 ± 0.08	2.43 ± 0.22	2.38 ± 0.05	2.34 ± 0.23
DD93-Erythromycin (50-1 mg/l)	5.55 ± 0.07	2.50 ± 0.2	2.54 ± 0.13	2.40 ± 0.07	2.54 ± 0.22


*Log_10_ colony count lower than that at time Zero without antimicrobial agent (control). -1 = Δ 1 Log_10_ CFU/ml = 90% killing; -2 = Δ 2 Log_10_ CFU/ml = 99% killing; -3 = Δ 3 Log_10_ CFU/ml = 99.9% killing.*

### Sequence Alignments and Blast Analysis

**FIGURE 3:**
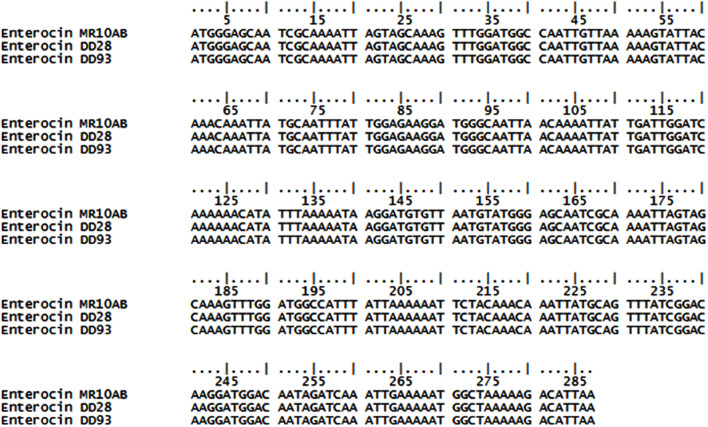
**Alignment of putative enterocins DD98 and DD93 DNA sequences using ClustalO (http://www.ebi.ac.uk/Tools/msa/) software.** (.) Sign indicates same nucleotide in the considered position for all the sequences aligned.

Amplification of total DNA from *E. faecalis* 28 and *E. faecalis* 93 with the reverse and forward primers previously used to amplify DNA coding for enterocins L50A and L50B (Cintas et al., 1998) permitted in the present study to obtain 287 bp DNA amplicons. Afterward, these amplicons were successfully cloned into the pGEM-T plasmid and sequenced using 7T and SP6 primers. The resulting sequences were blasted on blastn pubmed database and showed complete alignment with the sequences of enterocin MR10A and MR10B (**Figure 3**), which were two class IIb bacteriocins produced by *E. faecalis* MRR 10-3 (Martín-Platero et al., 2006).

### Biofunctionalization of Stainless Steel and Glass Slides on MRSA Biofilm Formation

**FIGURE 4:**
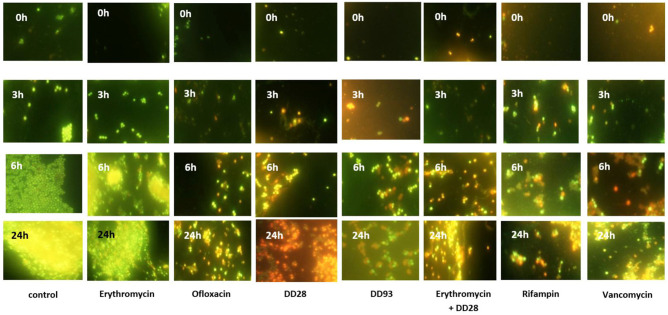
**Biofilms formation by MRSA-S1 on AISI 304L stainless steel slides conditioned with antimicrobial agents for 2 h and then washed and inoculated with 2 ml of 10^7^ CFU/ml MRSA-S1 culture before removing, washing and adding TSB-YE medium for 0, 3, 6, and 24 h of incubation at 37°C.** Concentrations of antimicrobial agents used were 8 mg/l for erythromycin, 0.03 mg/l for rifampin, 200 mg/L for enterocin DD28, 50 mg/l + 1 mg/l for enterocin DD28 + erythromycin, respectively. Biofilms were stained with the BacLight Live/Dead Viability Kit and imaged by epifluorescence microscopy after staining pattern for live cells (green) and dead cells (red). The experiments were performed at least twice and representative images are shown.

MRSA-S1 exhibited resistance to erythromycin. Thus, when the AISI 304L stainless steel slides were conditioned with 8 mg/l erythromycin, biofilm formation, as supported by epifluorescence microscopy, occurred normally. These results were comparable to those obtained with the untreated stainless steel slide (**Figure 4**; **Table 6**), advocating that MRSA-S1 biofilm formation was not affected by this antibiotic. Enterocins DD 28 or DD93 alone, at 200 mg/l, delayed the MRSA-S1 biofilm formation compared to the control test (**Figure 4**; **Table 6**). Remarkably, this result is similar to those obtained with vancomycin (1 mg/l) and rifampin (0.03 mg/l), for which the MRSA-S1 strain exhibited susceptibility (**Table 1**), and used as positive controls. Further, the combination of erythromycin and enterocin DD28 at 1 mg/l and 50 mg/l, respectively, has led to similar feature as that obtained with antibiotics.

**Table 6:** Effect of antimicrobials on MRSA-S1 biofilm formation at different incubation times on AISI 304L stainless steel slides.

Time (h)	0 h	3 h	6 h	24 h
Control	5.55 ± 0.08	7.25 ± 0.15	7.81 ± 0.18	8.85 ± 0.11
Erythromycin (8 mg/l)	5.25 ± 0.25	7.09 ± 0.19	7.39 ± 0.29	8.57 ± 0.12
Ofloxacin (0.5 mg/l)	3.12 ± 0.12	5.24 ± 0.22	5.58 ± 0.18	6.45 ± 0.13
Vancomycin (1 mg/l)	3.10 ± 0.08	5.29 ± 0.19	5.20 ± 0.14	6.10 ± 0.03
Rifampin (0.03 mg/l)	3.11 ± 0.10	5.17 ± 0.11	5.28 ± 0.17	6.06 ± 0.04
Enterocin DD28 (200 mg/l)	3.29 ± 0.10	5.51 ± 0.10	5.62 ± 0.13	6.54 ± 0.12
Enterocin DD93 (200 mg/ml)	3.35 ± 0.21	5.59 ± 0.10	5.58 ± 0.19	6.58 ± 0.17
Erythromycin / DD28 (1/50 mg/l)	3.35 ± 0.10	5.32 ± 0.17	5.15 ± 0.25	6.13 ± 0.10


*The data (Log_10_ CFU/ml) (±SD) are the means of at least three independent experiments.*

**FIGURE 5:**
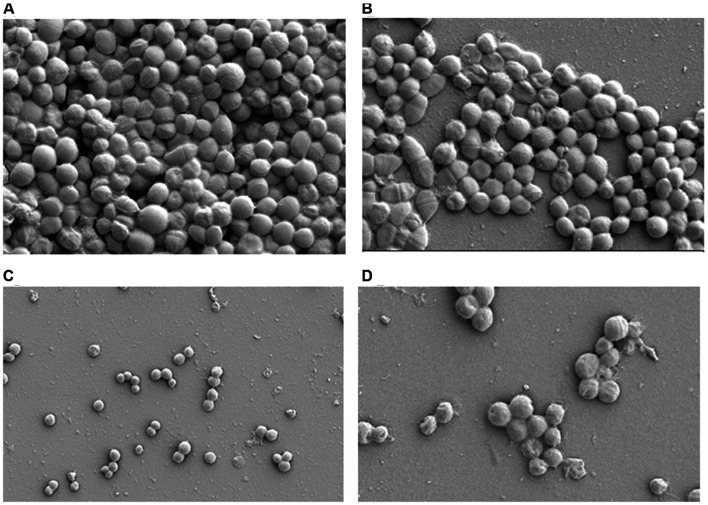
**Scanning electron microscope (SEM) of biofilms formation by MRSA-S1 strain on glass slides.** **(A)** Corresponds to the untreated sample (control) carrying 5 days old biofilm. **(B–D)** Show the samples conditionned with enterocin DD28 (200 mg/l), Erythromicin + enterocin DD28 (1–50 mg/l) and vancomycin (1 mg/l), respectively.

Scanning electron microscope microscopy analysis showed lower number of adherent MRSA-S1 cells on glass devices after conditioning with vancomycin (1 mg/l) and a combination of enterocin DD28+erythromycin (50/1 mg/l), comparatively to the unconditioned glass slide (**Figure 5**). However, the biofilm formation on a glass slide treated with enterocin DD28 alone (200 mg/l) showed more limited effect on the colonization of the surface compared to an untreated glass slide (**Figure 5**).

## Discussion

*Staphylococcus aureus* is the most often species encountered in the infectious diseases, generally as benign ones, but it can be involved in more serious infections as pneumonia, bacterial meningitis, and gastroenteritis. Moreover, this pathogen was also implied in the nosocomial infections, sometimes affecting bloodstream, joints, bones, lungs, or heart, resulting in very complicated infections, even with fatal epilog (Falcone et al., 2015). The methicillin-resistance was associated with a resistance to all β-lactams and some other antibiotics such as aminoglycosides and related macrolides, synergistines, fluoroquinolones, and fosfomycin. Some molecules including glycopeptides, rifampin, and fusidic acid remain active against MRSA (Hamdad et al., 2006; Falcone et al., 2015).

Staphylococci were shown to form biofilms on abiotic surfaces such as stainless steel, catheters and polystyrene material (Otto, 2008), and the biofilm lifestyle stands as a hurdle to antibiotic treatments that need to be overcome. The *icaADBC* genes coding for synthesis of exopolysaccharide of polymer matrix were shown to be involved in the biofilm formation (Tremblay et al., 2014). Biofilms act as a physical barrier, limiting drugs penetration. The ability of biofilm formation was also observed for other staphylococci species as *S. epidermidis* (Tremblay et al., 2014; Agarwal et al., 2015).

The present study permitted to shed light on the potential of enterocins DD28 and DD93 to help treating the MRSA burden. Furthermore, MRSA from hospital settings, are known to be resistant to erythromycin and kanamycin but remain sensitive to vancomycin and rifampin (Dumitrescu et al., 2010; Bhattacharya et al., 2015; Parhizgari et al., 2016).

Enterocins DD28 and DD93, two class IIb bacteriocins produced by *E. faecalis* DD28 and *E. faecalis* DD93 strains recently isolated from meconium were purified using the protocol described by Abriouel et al. (2003) and characterized for their masses and DNA sequences. Enterocins DD28 and DD93 masses were perfectly matching with those reported for enterocins enterocins L50A and L50B (Cintas et al., 2000), enterocins MR10A and MR10B (Abriouel et al., 2003) and enterocins A5–11A and A5–11B (Batdorj et al., 2006).

The anti-MRSA activity obtained with the semi-purified enterocins DD28 and DD93 appeared to be strain dependent. The highest MIC value was observed for the clinical MRSA-S1 strain, whilst the lowest ones were observed for *S. epidermidis* and *S. aureus* ATCC 33862. The anti-Staphylococcal activities obtained with enterocins DD28 and DD93 were less pronounced than the treatment afflicted by the antibiotics alone. Nevertheless, the combinations of these antibiotics with enterocins have revealed very promising therapeutic options. Indeed, bacteriocins were able to synergize, as supported by the FICI values and the killing curves experiments, the effects of antibiotics. The important drop of MRSA-S1 strain growth, when treated with enterocin DD28 or DD93 combined to erythromycin or kanamycin, occurred during the first 3 h of growth. Related to this, a drop of about 3 Log_10_ CFU/ml, was registered upon treatment of MRSA-S1 strain with the aforementioned antibacterial combinations. Based on this data, we assume that bacteriocins inputs for the anti-MRSA-S1 activity happened in the beginning of the treatment before their possible but plausible degradation. As the anti-MRSA-S1 activity was improved by the addition of the bacteriocins, and this strain could switch from a resistant to sensitive phenotype for the tested antibiotics, we support that anti-MRSA activity could be improved by incorporation of bacteriocins and the problem of their stability could be resolved by using encapsulated bacteriocins or nanoparticles coated bacteriocins.

Staphylococci were also reported to form biofilms on different biomaterials (Nan et al., 2015) designing them as guilty of recurrent infections taking place in the healthcare units (Amalaradjou and Venkitanarayanan, 2014). Mature biofilms are extremely difficult to eradicate (Dosler and Mataraci, 2013) complicating thereof treatment of this pathogen. The conditioning of AISI 304L stainless steel and glass slides, with enterocin DD28 and antibiotics impeded the biofilm formation by the MRSA-S1 strain. The combinations of these antimicrobials has not only impacted the growth of the MRSA-S1 strain under planktonic culture but hampered the biofilm formation. The MRSA-S1 strain population was reduced of about 2 Log_10_ CFU/ml, on AISI 304L stainless steel slides. This data was logically correlated to observations resulting from the epifluorescence and SEM analyses.

## Conclusion

This study permitted to shed light on the anti-MRSA activity of enterocins DD28 and DD93. Importantly, these bacteriocins were able to synergize with erythromycin and kanamycin, two antibiotics used in the MRSA treatment. The data gathered in the frame of this work enabled us to confirm the role of bacteriocins as a novel class of antibiotics to assist or replace the fading antibiotics. The perfect similarities exhibited by DNA and amino-acids sequences of enterocins DD28 and DD93 vs. enterocins MR10A and MR10B let us to think that these bacteriocins are most probably similar.

## Author Contributions

SS and RB performed the SEM analysis of MRSA-S1 strain grown on glass (treated or not with antimicrobials). AV realized the Vitek-2antibiograms and provided us with the *S. epidermidis* strain used as well in this work. AA realized most of the experiments suchas MIC values, Killing curves, biofilms assays on steel slides and glass. AA did also the epifluorescence imaging YB contributed to MIC determinations and DNA sequences analyses GC realized the mass analysis of enterocins DD28 and DD93, RR and DD supervised this work. All authors read and approved the manuscript.

## Conflict of Interest Statement

The authors declare that the research was conducted in the absence of any commercial or financial relationships that could be construed as a potential conflict of interest.
