# Correction: Anti-MRSA activities of enterocins DD28 and DD93 and evidences on their role in the inhibition of biofilm formation

**DOI:** 10.3389/fmicb.2025.1646639

**Published:** 2025-08-11

**Authors:** Ahmed K. Al Atya, Yanath Belguesmia, Gabrielle Chataigne, Rozenn Ravallec, Anne Vachée, Sabine Szunerits, Rabah Boukherroub, Djamel Drider

**Affiliations:** ^1^Université de Lille 1 Sciences et Technologies - Institut Charles Viollette, Lille, France; ^2^Hôpital Victor Provo de Roubaix, Roubaix, France; ^3^Institut d'Electronique, de Microélectronique et de Nanotechnologie, UMR CNRS 8520, Université Lille 1, Lille, France

**Keywords:** enterocins, antibiotics, MRSA, synergism, bacteriocins

There was a mistake in Figure 4 as published. A wrong figure appeared.

The corrected Figure 4 and it's caption appear below.

**Figure 4:**
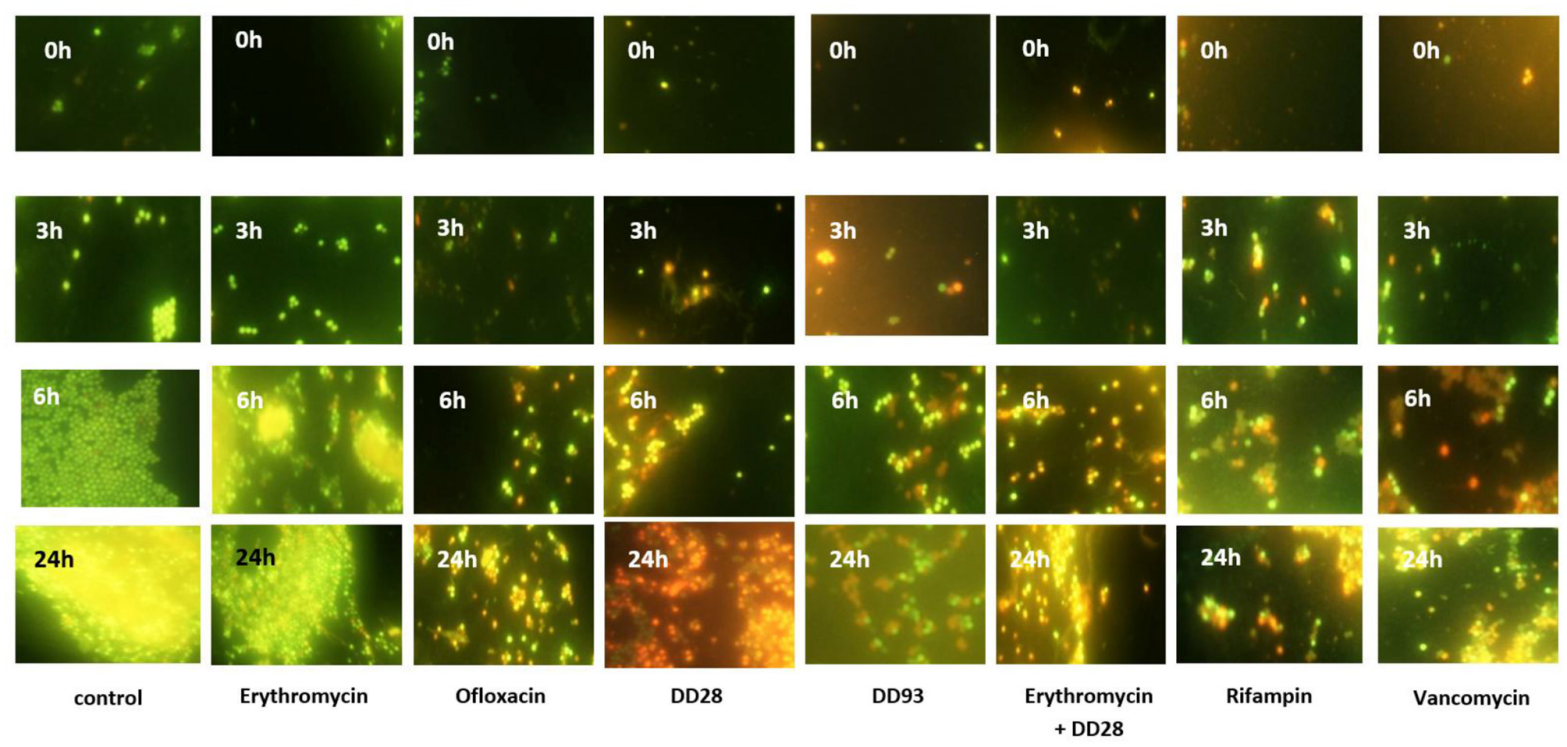
Biofilms formation by MRSA-S1 on AISI 304L stainless steel slides conditioned with antimicrobial agents for 2 h and then washed and inoculated with 2 ml of 10^7^ CFU/ml MRSA-S1 culture before removing, washing and adding TSB-YE medium for 0, 3, 6, and 24 h of incubation at 37°C. Concentrations of antimicrobial agents used were 8 mg/L for erythromycin, 0.03 mg/L for rifampin, 200 mg/L for enterocin DD28, 50 mg/L + 1 mg/L for enterocin DD28 + erythromycin, respectively. Biofilms were stained with the BacLight Live/Dead Viability Kit and imaged by epifluorescence microscopy after staining pattern for live cells (green) and dead cells (red). The experiments were performed at least twice and representative images are shown.

The original version of this article has been updated.

